# Exome screening to identify loss-of-function mutations in the rhesus macaque for development of preclinical models of human disease

**DOI:** 10.1186/s12864-016-2509-5

**Published:** 2016-03-02

**Authors:** Adam S. Cornish, Robert M. Gibbs, Robert B. Norgren

**Affiliations:** Department of Genetics, Cell Biology and Anatomy, University of Nebraska Medical Center, Omaha, 68198-5805 Nebraska

**Keywords:** Rhesus, Macaca mulatta, Macaque, Exome, Mutation, Loss-of-function, BChE, RNASEL, Null mutant, Cancer

## Abstract

**Background:**

Exome sequencing has been utilized to identify genetic variants associated with disease in humans. Identification of loss-of-function mutations with exome sequencing in rhesus macaques (*Macaca mulatta*) could lead to valuable animal models of genetic disease. Attempts have been made to identify variants in rhesus macaques by aligning exome data against the rheMac2 draft genome. However, such efforts have been impaired due to the incompleteness and annotation errors associated with rheMac2. We wished to determine whether aligning exome reads against our new, improved rhesus genome, MacaM, could be used to identify high impact, loss-of-function mutations in rhesus macaques that would be relevant to human disease.

**Results:**

We compared alignments of exome reads from four rhesus macaques, the reference animal and three unrelated animals, against rheMac2 and MacaM. Substantially more reads aligned against MacaM than rheMac2. We followed the Broad Institute’s Best Practice guidelines for variant discovery which utilizes the Genome Analysis Toolkit to identify high impact mutations. When rheMac2 was used as the reference genome, a large number of apparent false positives were identified. When MacaM was used as the reference genome, the number of false positives was greatly reduced. After examining the variant analyses conducted with MacaM as reference genome, we identified two putative loss-of-function mutations, in the heterozygous state, in genes related to human health. Sanger sequencing confirmed the presence of these mutations. We followed the transmission of one of these mutations (in the butyrylthiocholine gene) through three generations of rhesus macaques. Further, we demonstrated a functional decrease in butyrylthiocholinesterase activity similar to that observed in human heterozygotes with loss-of-function mutations in the same gene.

**Conclusions:**

The new MacaM genome can be effectively utilized to identify loss-of-function mutations in rhesus macaques without generating a high level of false positives. In some cases, heterozygotes may be immediately useful as models of human disease. For diseases where homozygous mutants are needed, directed breeding of loss-of-function heterozygous animals could be used to create rhesus macaque models of human genetic disease. The approach we describe here could be applied to other mammals, but only if their genomes have been improved beyond draft status.

**Electronic supplementary material:**

The online version of this article (doi:10.1186/s12864-016-2509-5) contains supplementary material, which is available to authorized users.

## Background

The study of genetic variants associated with disease is revolutionizing biomedical research and promises to make profound changes in clinical practice [1–4]. One limitation to current approaches has been the high cost of genome sequencing. However, next generation sequencing (NGS), which can focus on the “exome” (the sequences contained in exons), offers a less expensive method. Already, exome sequencing has been used with great success to identify genomic sequence variants associated with disease in humans [2, 4–6].

Identifying a genetic variant associated with a human disease is an important first step, but animal models that match human phenotypes are necessary to develop effective therapeutics. Although mouse models have been helpful in understanding basic biology, their great evolutionary distance and biological differences from humans can limit their utility for preclinical studies [7]. Approximately 90 % of therapeutic drug candidates fail to advance during the clinical phases of pharmaceutical development [8]. The lack of good animal models for efficacy testing is likely one cause of this high failure rate.

Rhesus macaques (*Macaca mulatta*) and other nonhuman primates are similar to humans in both physiology and anatomy due to their close evolutionary relationship [9]. They have proven essential as models for infectious diseases such as AIDS/HIV and neurological and reproductive disorders [10–16].

Rhesus macaques with genetic variations similar to those that cause disease in humans might prove invaluable as preclinical models. A rhesus macaque model of Huntington’s disease has been created by inserting a Huntingtin gene with a pathological number of repeats into the genome of oocytes using lentiviral vectors [17]. This gain-of-function mutation has been shown to result in a phenotype similar to human Huntington’s patients [18]. Recently, the CRISPR/Cas9 system has been used to genetically modify the genome of a cynomolgus macaque (*Macaca fascicularis*) [19]. Although these approaches have much promise, they require advanced in vitro fertilization facilities which are very limited for nonhuman primates.

Another potential approach to providing rhesus macaques models of genetic disease would be to examine the exomes of “normal” rhesus macaques for high impact mutations. Each individual human has, on average, three to five recessive mutations in genes related to Mendelian disorders. This statistic was first obtained from studies of consanguineous marriages [20] and later confirmed with exome analyses [1]. A study that compared SNPs in the hippocampal RNA sequences of humans and rhesus macaques found that the number of damaging coding variations was similar in the two species [21]. Assuming a similar incidence of loss-of-function (LOF) mutations in rhesus macaques as is found in humans, exome sequencing and analyses could be used to create a catalog of mutations in genes related to Mendelian disorders in large numbers of animals. Heterozygotes may be immediately useful. However, if homozygous individuals are required, heterozygotes with LOF mutations could be bred together to produce null mutant offspring [9].

Exome analysis requires two important resources: exome capture probes that can capture genomic fragments containing exons and a high quality reference genome against which sequences can be aligned. For humans and mice, these resources exist. For rhesus macaques, no species-specific exome capture probe sets are available. Fortunately, two previous studies have shown that human probes can be used to capture about 96 % of the rhesus macaque coding exons with a minimum of 1X coverage [22, 23]. However, until recently, investigators interested in performing exome analyses in rhesus macaques were limited to using a draft rhesus genome, rheMac2, which contained many errors and was incomplete [24]. Serious issues related to using this genome for exome analyses have been reported [23]. We have recently completed a high quality reference genome for the rhesus macaque, MacaM [25]. Here, we show that alignments of exome sequences with MacaM can be analyzed to identify LOF mutations in rhesus macaques related to human disease.

## Methods

We used two human exome capture kits to enrich for exons in four rhesus macaques: the TruSeq Exome Enrichment Kit (Illumina) for animal 002 T-NHP and the SureSelect XT HumanAllExon 50 Mb Kit (Agilent, G7544A) for animals 17573, ON12033 and ON22186.

We sequenced exomic fragments with an Illumina Genome Analyzer IIx (for animal 002 T-NHP) and a HiSeq2000 (for animals 17573, ON12033 and ON22186) and obtained 101 bp paired-end reads for each exome generating a total of 17.7 Gb of data. We deposited sequences in the Sequence Read Archive at the National Center for Biotechnology Information (NCBI) under accessions SRX144674 (002 T-NHP), SRX115899 (animal 17573), SRX144808 (animal ON12033) and SRX145282 (animal ON22186).

We followed The Broad Institute’s Best Practices guidelines for discovering putative variants utilizing the Genome Analysis Toolkit (GATK) [26] to identify LOF mutations. Briefly, we aligned exomic sequences from the four rhesus macaques to the two different reference rhesus assemblies to be analyzed: rheMac2 (downloaded from the UCSC Genome Browser on June 24, 2014) and MacaM (version 7) using bwa mem (version 0.7.5a-r405). Preliminary analyses indicated that aligning exome reads against only chromosome files resulted in apparent misalignments of pseudogene reads against coding genes. When unplaced and unlocalized scaffolds were included in the reference assembly, misalignments involving coding genes were reduced. All data reported in the results for both rheMac2 and MacaM were collected using the latter approach. We processed the 8 alignment files (four samples x two genomes) to remove PCR duplicates (using Picard Tools version 1.137), realign around putative indels (using GATK version 3.4), and re-calibrate quality base scores (using GATK version 3.4). We used the Samtools (version 0.1.18) flagstat tool to obtain the “percent aligned” for each of these datasets (Table 1).

**Table 1:** Percent of rhesus exome reads aligned against different assemblies

Sample ID	Sample accession	rheMac2 assembly chr only	MacaMv7 assembly chr only	rheMac2 assembly chr + unplaced	MacaMv7 assembly chr + unplaced
17573	SRX115899	94.28 %	97.03 %	96.74 %	97.57 %
ON12033	SRX144808	93.19 %	96.29 %	95.98 %	96.95 %
ON22186	SRX145282	92.62 %	96.21 %	95.84 %	96.95 %
002T-NHP	SRX144674	92.09 %	95.12 %	94.87 %	95.84 %

*Chr only* alignments against only scaffolds placed on chromosomes; *Chr + unplaced* alignments against both placed and unplaced scaffolds

We identified putative variants in the processed alignment files with the GATK HaplotypeCaller (version 3.4). We removed low quality variants using The Broad Institute’s recommended filters [27]. For SNPs, we removed variants that had a Depth (DP) < 5, QualByDepth (QD) score < 2.0, FisherStrand (FS) score > 60.0, RMSMappingQuality (MQ) < 40.0, MappingQualityRankSum Test (MQRankSum) score < −12.5, or a ReadPosRankSumTest (ReadPosRankSum) score < −8.0. For insertions and deletions, we removed variants that had a DP < 5, QD < 2.0, FS > 200.0, or ReadPosRankSum < −20.0. We used snpEff (version 3.1 h) [28] to generate a tab-delimited file with variant information to identify putative LOF mutations in both genomes. We obtained the rheMac2 annotation files from the NCBI ftp genome database on July 20, 2015. For MacaM, we used annotation version 7.6 [25]. We focused on mutations expected to cause complete LOF of protein, specifically premature stop codon and frameshift mutations. We used the Online Mendelian Inheritance in Man database [29] to select genes known to be involved in disease in humans. We manually inspected candidate mutations in these genes with the Integrated Genome Viewer (IGV) [30]. We did not further investigate variants that did not seem likely to result in a high impact mutation. Coding variant statistics were obtained using SnpEff following filtering and annotation.

We designed PCR primers with Primer3 [31] to flank target exons containing candidate mutations in two genes, butyrylcholinesterase (BChE) and ribonuclease L (RNASEL). We included M13F (−20) sequence (GTAAAACGACGGCCAGT) and M13R sequence (GGAAACAGCTATGACCATG) on the 5' end of the primer sequences to facilitate Sanger sequencing.

Primer sequences:

- BChE (forward): GTAAAACGACGGCCAGTGGGGACAACAAATGCTTCAT
- BChE (reverse): GGAAACAGCTATGACCATGTGGAACCCAAACACTGACCT
- RNASEL (forward): GTAAAACGACGGCCAGTGGGCCATATACTGCCTTGAA
- RNASEL (reverse): GGAAACAGCTATGACCATGATTCTGCATGATGGGAGAGG

Integrated DNA Technologies (Coralville, Iowa) synthesized the primers. We performed PCR with these primers and genomic DNA from two animals from the Oregon National Primate Research Center, ON12033 (RNASEL) and ON22186 (BChE). Briefly, 200 ng of genomic DNA was used as the template for PCR using the AccuPrime Pfx Supermix Kit (Invitrogen) according to the manufacturer’s instructions. We amplified targeted exons in a MJ Research PTC-100 thermocycler with the following program:

- Step 1. Denature at 95 °C for 5 minutes.
- Step 2. For 35 cycles: 95 °C for 15 seconds; 60 °C for 30 seconds; 68 °C for 45 seconds.

We purified PCR products with the QIAquick PCR Purification Kit (Qiagen) and then sequenced the PCR products with the Sanger method. We manually inspected traces to identify putative mutations.

We performed PCR for BChE on genomic DNA from descendants of ON22186 including: ON22187, ON22188, ON22191, ON22192 and ON22193.

To determine whether an identified LOF mutation in the BChE gene (in the heterozygous state) resulted in a decrease in BChE activity, a functional assay for this enzyme was performed. 4 ml of heparinized whole blood was drawn from two animals at the Oregon National Primate Research Center. One animal, ON22193, had been identified with a LOF mutation (in the heterozygous state) in the BChE gene. Animal ON22197 was an unrelated cage mate. Both animals were female.

The whole blood was transferred to sterile microfuge tubes using aseptic conditions. 0.4 ml was placed in a nonsterile tube for activity assays. Tubes were centrifuged for 10 min to pellet the red blood cells. The red blood plasma was then transferred to new tubes.

BChE activity was measured in 0.1 M potassium phosphate buffer pH 7.0 containing 0.5 mM dithiobisnitrobenzoic acid, and 1 mM butyrylthiocholine at 25 °C. The increase in absorbance was recorded at 412 nm and converted to μmoles butyrylthiocholine hydrolyzed per min using the extinction coefficient 13,600 M^−1^ cm^−1^ [32]. Units of activity were measured in μmoles per minute. Each plasma sample was assayed in triplicate.

Two specific BChE inhibitors, 20 μM ethopropazine, and 0.1 mM iso-OMPA were used to demonstrate that the measured activity was catalyzed by BChE. Plasma was preincubated with 0.1 mM iso-OMPA for 30 minutes before the reaction was started by addition of 0.02 ml of 0.2 M butyrylthiocholine.

### Statement of ethical approval

Materials used in these studies were from animal work performed under Institutional Animal Care and Use Committee approval from the University of Nebraska Medical Center and Oregon Health and Sciences University. Animal welfare was maintained by following NIH (Public Health Service, Office of Laboratory Animal Welfare) and USDA guidelines by trained veterinary staff and researchers under Association for Assessment and Accreditation of Laboratory Animal Care certification, insuring standards for housing, health care, nutrition, environmental enrichment and psychological well-being. These met or exceeded those set forth in the Guide for the Care and Use of Laboratory Animals from the National Research Council of the US National Academy of Sciences.

## Results

We aligned exome capture reads from four rhesus macaques against rheMac2 and MacaM (Table 1). Substantially more reads aligned against MacaM than rheMac2 when reads were aligned only against scaffolds placed on chromosomes. When unplaced scaffolds were used, the differences in alignment percentages between rheMac2 and MacaM were greatly reduced.

We used GATK HaplotypeCaller to identify potentially high impact mutations in the four rhesus macaque exomes using the rheMac2 and MacaM assemblies (Table 2). Substantially more LOF mutations were called when exome sequences were aligned against rheMac2 than MacaM. One way to determine whether these calls are accurate is to examine putative homozygous mutations identified in the reference animal, 17573. Since sequences from 17573 were used to create both the rheMac2 and MacaM assemblies, no homozygous mutations should be called when exome sequences from this animal are aligned against the reference assemblies.

**Table 2:** Variant calls for exomes aligned against rheMac2 and MacaM7 assemblies

	rheMac2	rheMac2	MacaM7	MacaM7
Variant	Het	Hom	Het	Hom
Animal 17573				
START_LOST	34	4	8	0
STOP_GAINED	200	25	42	0
STOP_LOST	86	70	5	0
FRAME_SHIFT	394	669	100	4
SPLICE_SITE_ACCEPTOR	59	49	27	0
SPLICE_SITE_DONOR	70	38	28	3
Animal ON12033				
START_LOST	36	19	13	4
STOP_GAINED	278	84	99	9
STOP_LOST	71	132	6	3
FRAME_SHIFT	618	722	232	29
SPLICE_SITE_ACCEPTOR	74	68	45	11
SPLICE_SITE_DONOR	91	71	50	13
Animal ON22186				
START_LOST	29	20	9	4
STOP_GAINED	211	77	59	6
STOP_LOST	68	116	10	0
FRAME_SHIFT	484	778	188	24
SPLICE_SITE_ACCEPTOR	69	64	33	14
SPLICE_SITE_DONOR	73	60	34	13
Animal 002 T-NHP				
START_LOST	25	18	10	2
STOP_GAINED	222	67	86	5
STOP_LOST	55	112	10	1
FRAME_SHIFT	505	654	228	27
SPLICE_SITE_ACCEPTOR	77	57	52	6
SPLICE_SITE_DONOR	84	60	48	10

In general, our results suggest a high false positive rate when rheMac2 is used as the reference genome. For example, with rheMac2, 610 homozygous frameshifts were called in animal 17573 compared to 350 heterozygous frameshifts (Table 2). This pattern of mutation is extremely unlikely from a biological perspective. When we used MacaM as the reference genome, we observed 92 heterozygous and 4 homozygous frameshifts, suggesting a much lower false positive rate with this rhesus macaque genome. A similar pattern of higher false positives with the rheMac2 genome than with the MacaM genome was also observed for other types of mutations (Table 2).

**Fig. 1:**
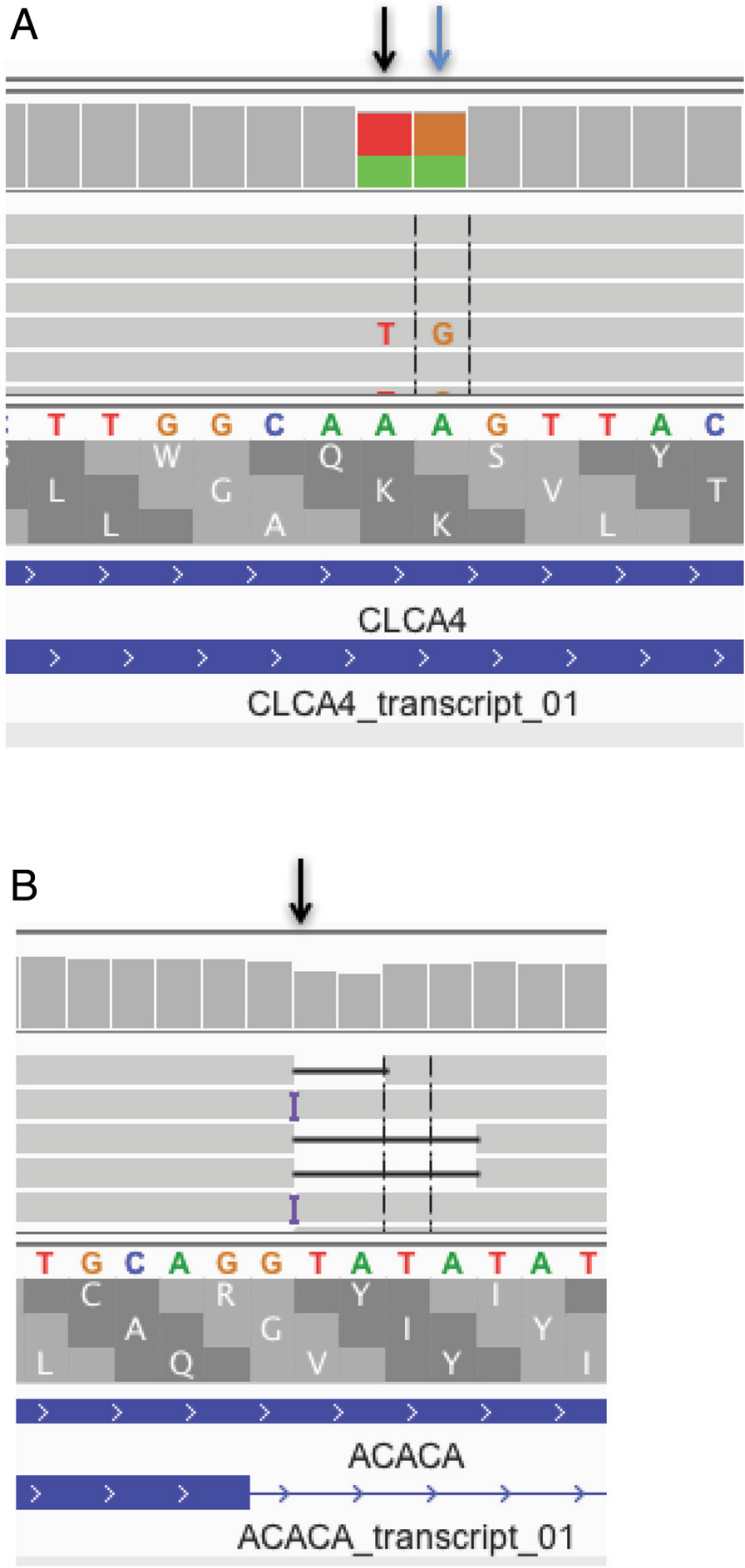
Mutations identified as “high impact” by SnpEff but which likely have no impact. **a**. An A > T mutation at position 86408706 of chromosome 1 (black arrow) was identified as a premature stop codon by SnpEff. If this mutation had happened in isolation, it would in fact result in p.Lys374* in the CLCA4 protein (bottom frame), as predicted by SnpEff. However, because there was also an A > G mutation in the adjacent nucleotide (blue arrow), the actual change would be p.Lys374Trp, a missense, not a LOF mutation. **b**. A “TA” insertion at position 30707229 of chromosome 17 (black arrow) was identified as a “high impact” splice_donor_variant by SnpEff. In fact, this insertion would leave a “GT” donor intact. It would simply replace one “T” for another. It is also possible that HaplotypeCaller had difficulty with the alignments in this region due to “TATA” repeats. For both 1A and 1B, mutations are reported for animal 17573 using the MacaM genome. Figures are screenshots of alignments viewed with IGV.

Although there were many fewer false positives with MacaM than rheMac2, it was still necessary to filter the output from SnpEff to focus on the candidate variants most likely to have a high impact. For example, apparent variants that were found in multiple, unrelated animals were considered unlikely to be high impact. We found that manual inspection of putative high impact mutations in IGV was helpful in understanding why this was so. In some cases, snpEff reported variants which, when considered in isolation, would cause high impact mutations, but when considered in context, did not cause a significant change in the genome. For example, a change in one nucleotide reported to result in a premature stop codon in the chloride channel accessory 4 (CLCA4) gene actually did not cause a premature stop codon because an adjacent nucleotide change prevented a stop codon from being produced (Fig. 1a). In another case, a reported disruption of the splice donor site by the insertion of “TA” after the “G” in a splice donor site of the acetyl-CoA carboxylase alpha (ACACA) gene would in fact would still result in an intact “GT” (Fig. 1b).

Incorrect alignments also appeared to be responsible for incorrect calls. In preliminary experiments, we aligned exome reads against chromosome files alone. We noted a significant numbers of “high impact” calls in regions with apparent very high polymorphism and allele ratios that were far from 1:1. We hypothesized that the exome capture kits had pulled down pseudogene fragments and that because pseudogenes had not been included in our chromosome assembly these fragments were aligned against coding genes similar in sequence to pseudogenes thus creating false positive signals. To test this hypothesis and attempt to decrease false positives, we redid our analyses, this time including unplaced scaffolds in the alignment step. Our reasoning was that pseudogene exome fragments would now align against pseudogene loci in the unplaced scaffolds rather than against protein coding genes thereby reducing the number of false positives due to misalignments. In fact, we did observe a dramatic reduction in such false positives after including unplaced scaffolds for alignments [data not shown]. We also observed that there were many more apparent false positives due to misalignment in animal 002T-NHP than the other three animals [data not shown]. It may be that the TruSeq Exome Enrichment Kit, which was used only for this animal, contained probes which were more likely to hybridize to pseudogene fragments than the the SureSelect XT HumanAllExon 50 Mb Kit, which was used for the other three animals.

Exome analysis using MacaM as the reference identified many variants (Additional file 1: Table S1). As expected, mutations in noncoding regions were much more common than mutations in coding regions (especially nonsense mutations) (Table 3). As a percent of total SNPs located in the CDS, synonymous SNPs constituted 64.6 to 65.7 % of the total, non-synonymous SNPs constituted 34.1 to 35.2 % of the total and nonsense SNPs constituted 0.2 to 0.3 % of the total (Table 3). We also examined splice sites for exons containing coding sequence (CDS) and untranslated regions (UTR) (Additional file 2: Table S2). Mutations at splice sites were rare for all exons suggesting that both types of junctions are under negative selection.

**Table 3:** SNPs located in CDS

	17573	ON12033	ON22186	002 T-NHP
Synonymous	15,689 (64.6 %)	30,566 (65.7 %)	23,145 (64.9 %)	25,066 (65.0 %)
Non-synonymous	8563 (35.2 %)	15,874 (34.1 %)	12,416 (34.8 %)	13,399 (34.7 %)
Nonsense	50 (0.2 %)	109 (0.2 %)	100 (0.3 %)	99 (0.3 %)
Total	24,302	46,549	35,661	38,564

**Fig. 2:**
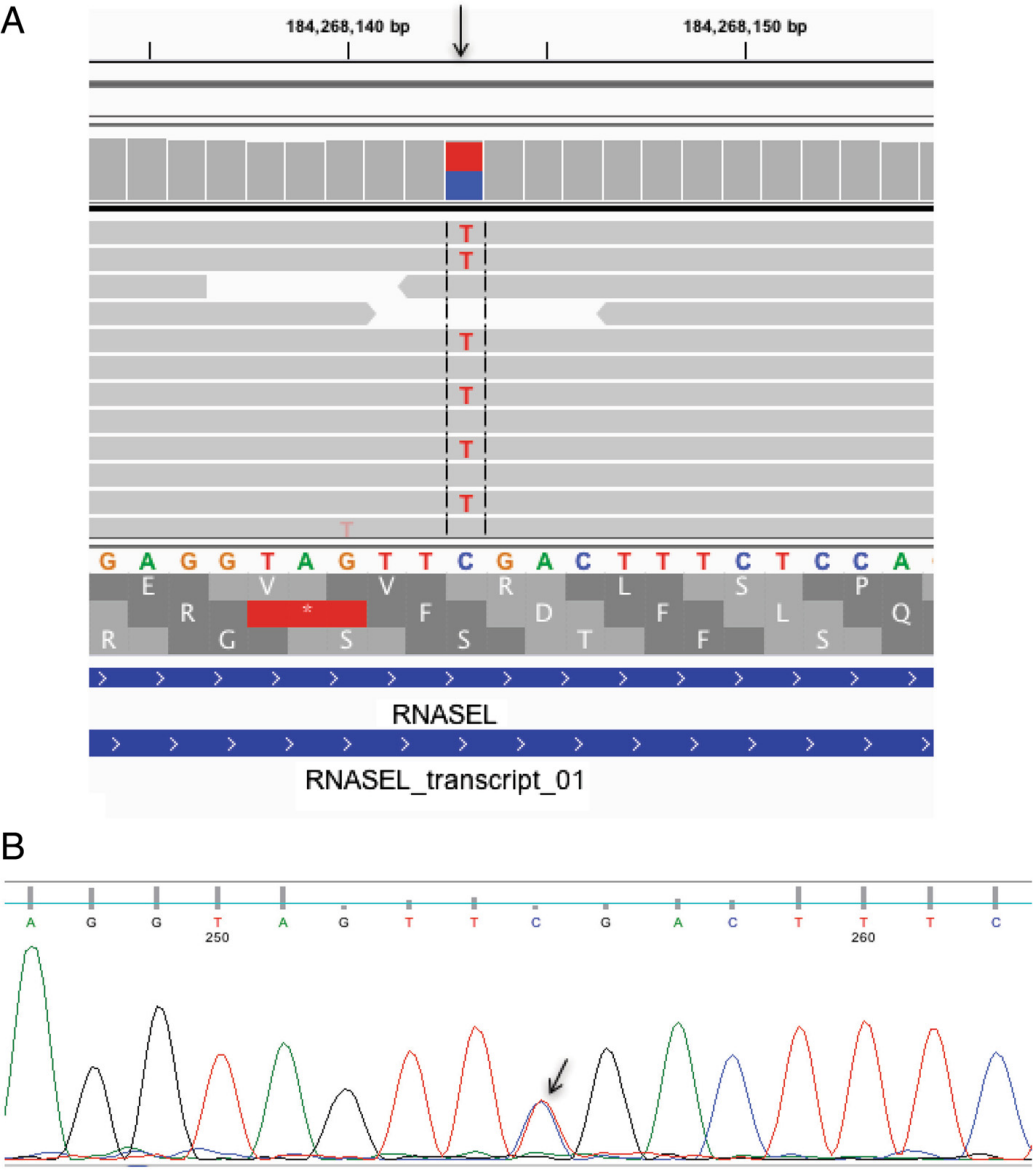
LOF premature stop codon mutation in the RNASEL gene in animal ON12033. **a**. IGV screenshot of mutation (in the heterozygous state) in the RNASEL gene in position 184268143 of chromosome 1 (arrow). The top frame is correct. The C > T mutation results in p.Arg552* in animal ON12033. **b**. Sanger sequence trace indicating the premature stop codon RNASEL c.1654C > T mutation in the heterozygous state (arrow) in animal ON12033

**Fig. 3:**
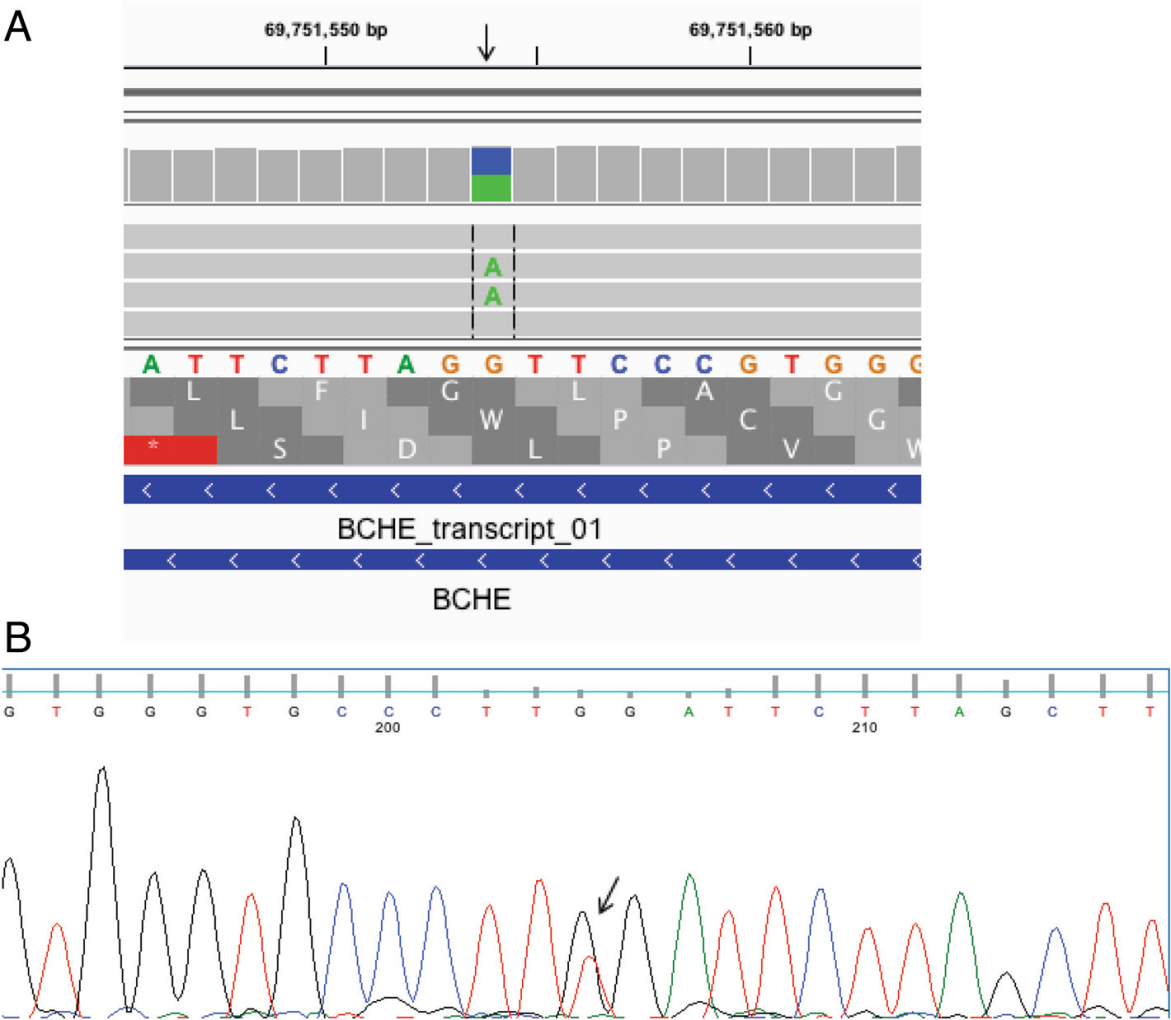
LOF premature stop codon mutation in the BChE gene. **a**. IGV screenshot of mutation (in the heterozygous state) in the BChE gene in position 69751554 of chromosome 3 (arrow). Because the gene is running in the antisense direction, the reference sequence should be read right to left. The aligned sequences are not reverse complemented so “C” = “G” and “A” = “T” with respect to the reference sequence. The top frame is correct. The G > T mutation results in p.Gly180*. This figure depicts exome sequence alignments for animal ON22186, the original animal in which the BChE mutation was detected. **b**. Sanger sequence trace indicating the premature stop codon BChE c.538G > T mutation in the heterozygous state (arrow) in animal ON22193, a third generation descendant of animal ON22186

**Fig. 4:**
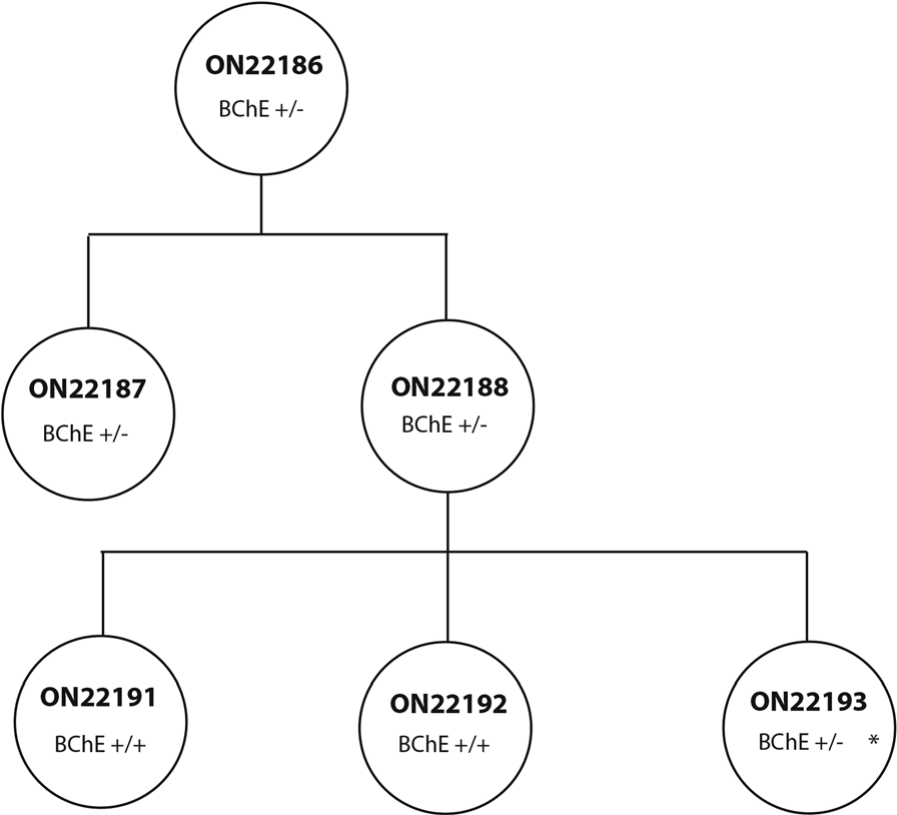
BChE transmission across three generations of rhesus macaques. * indicates that animal ON22193 was used for the BChE activity assay. This is also the animal whose BChE mutation is depicted in Fig. 3b.

We focused our analysis on two apparent high impact mutations in genes known to be related to human genetic disease, ribonuclease L (RNASEL) in animal ON12033 (chr01: 184268143) and BChE in animal ON22186 (chr03:69751554). Both mutations were annotated as “STOP-GAINED” in the heterozygous state by SnpEff with high confidence scores. The raw QUAL scores for these two mutations were 2755.77 and 1186.77, respectively. The mutation in the RNASEL gene (p.R552X) occurred in the fourth exon. This mutation was visualized with IGV (Fig. 2a) and validated with Sanger sequencing (Fig. 2b). The mutation in the BCHE gene (p.G180X) occurred in the second exon. This mutation was also visualized with IGV (Fig. 3a) and validated with Sanger sequencing (Fig. 3b). We determined the transmission of BCHE in a family of rhesus macaques using Sanger sequencing of exon 2 (Fig. 4). This mutation was transmitted across three generations (Fig. 4).

To determine whether the LOF mutation in BChE in the heterozygous state decreased BChE activity in rhesus macaques as it would in humans, we collected blood from animal ON22193 (a third generation carrier of the mutation) and animal ON22197 (an unrelated cage mate). Analysis of the serum of these two animals revealed that the heterozygous mutant had 30 % of the BChE activity of its unrelated cage mate (Table 4).

**Table 4:** Butyrlcholinesterase activity (units/ml) in a BChE LOF heterozygote (ON22193) and wild type cage mate (ON22197)

Animal ID	BChE activity - No inhibitor	BChE activity - 20 μM ethopropazine	BChE activity - 0.1 mM iso-OMPA
ON22193	1.38	0	0
ON22197	4.65	0	0

## Discussion

We have previously demonstrated that MacaM yields better results than rheMac2 for mRNA-sequence expression analysis [25, 33]. We now demonstrate that MacaM also performs better than rheMac2 for exome analysis. More exome reads align against the MacaM chromosome assembly than the rheMac2 assembly (Table 1). This gap largely disappears when unplaced scaffolds are included for the alignments (Table 1) suggesting that more of the rhesus genome is represented in the MacaM chromosome assembly than in the rheMac2 chromosome assembly. Further, MacaM has many fewer false positives than rheMac2 (Table 2). Although it is tempting to aggressively annotate as many genes as possible, for exome studies the cost of incorrectly annotating genes is very high. This is because an unacceptable number of false positive high impact mutations will result from these errors. This is likely in part due to the fact that the Gnomon automated annotator used by NCBI will use intronic sequence when an exon for a gene is missing from the chromosome assembly [24]. Since introns are often poorly conserved, attempts to align sequence against spurious “exons” (actually intronic sequence) will frequently result in apparent highly damaging mutations. The most time-intensive step in exome studies is filtering false-positives. Adding large numbers of false-positives as a result of incorrect gene annotations has made using the rhesus annotations for rheMac2 for exome studies in rhesus macaques impractical [23].

The overall number of SNPs and percent nonsense mutations that we observed for rhesus macaques are similar to those reported for humans [5]. The relative number of synonymous to non-synonymous SNPs, about 2 to 1, that we observed in rhesus macaques was strikingly similar to the ratio reported by Yuan et al. for rhesus macaque [21] but different from the approximately 1 to 1 ratio found in humans [5]. Negative selection is apparently acting in both species to limit the number of deleterious mutations.

Our results suggest that the new MacaM genome can be used with exome data to screen rhesus macaques for naturally occurring LOF mutations in genes related to human disease. We describe LOF mutations, in the heterozygous state, in two genes related to human health, RNASEL and BChE.

LOF mutations in RNASEL have been linked to susceptibility to prostate cancer in humans [34, 35]. Individuals with LOF germline mutations in the heterozygous state developed prostate tumors with complete loss of function (loss of heterozygosity) [34, 35]. Rhesus macaque heterozygotes with LOF mutations in the RNASEL gene might serve as important animal models for prostate cancer.

Humans with mutations in the BChE gene that decrease or eliminate expression of butyrylcholinesterase have prolonged apnea in response to exposure to succinylcholine or mivarcurium [36]. Further, treatment with BChE has been shown to be protective against a nerve agent, Soman, in rhesus macaques [37] indicating the possible importance of the levels of this enzyme in the likelihood of surviving exposure to nerve agents. We demonstrated that a rhesus macaque with a LOF mutation in the BChE gene in the heterozygous state had decreased BChE activity compared to an unrelated cagemate. Rhesus macaques with LOF mutations in the BChE gene may serve as useful models for pharmacogenomics and perhaps in the evaluation of the effects of nerve agents in individuals with varying levels of BChE activity.

Rare mutations are more likely to be present in the heterozygous state. For some diseases, the heterozygous mutants themselves would be useful. For Mendelian recessive genetic diseases, heterozygotes could be bred together to increase the numbers of homozygote null mutant rhesus macaques. In this way, nonhuman primate models of LOF human genetic disease could be produced. Given that there are tens of thousands of macaques at primate centers and at private facilities and that every animal likely harbors one or more interesting mutations, models for many genetic diseases could likely be created with a program of exome screening, genotyping and directed breeding.

The exome screening and directed breeding approach suggested here could provide a much-needed alternative to mouse models of human genetic disease. Given the many similarities in physiology and behavior between monkeys and humans, macaque models of genetic disease have the potential to transform preclinical research by providing greatly improved tests of the effectiveness of therapeutic agents.

Our work also provides motivation to improve other draft genomes. Other mammalian species, for which there are currently only draft genomes, could provide useful animal models for human disease using the exome screening and directed breeding approach we suggest here; but only if their genomes have been upgraded to MacaM level quality.

There has been great interest in CRISPR/Cas9 and other targeted approaches to producing null mutant animals in species other than mice. The advantage of these methods is that one can choose whichever target one wishes. However, they require use of assisted reproductive technologies (ARTs). For nonhuman primates, there are relatively few centers that have the necessary equipment and expertise to produce targeted null mutant animals due to limitations in access to the ARTs. The advantage of our suggested approach is that exome sequencing and conventional breeding are within the reach of most centers.

There are disadvantages to screening for naturally occurring mutations. Some genetic diseases are caused by rare *de novo* mutations (gain-of-function or dominant negative loss-of-function) that are not compatible with reproduction. In these cases, it would be difficult to produce a line of animals to study (although the animal with the *de novo* mutation could be examined and exome sequencing of this animal would reveal the molecular basis of its likely obvious phenotype). For many Mendelian recessive diseases, homozygous LOF animals would not be able to reproduce, so maintenance of unaffected lines of male and female heterozygous LOF animals would be required to produce new homozygotes. Another disadvantage to our approach is that one does not know in advance which mutations one will find. It is likely that each center which houses nonhuman primates will have its own set of mutations due to the founder effect and restricted breeding policies. The more animals and centers that are screened, the greater the number potential animal models that will be identified. In principal, if enough animals are screened, eventually LOF mutations in every gene related to disease in humans will be identified.

In the current work, we focused on unequivocal loss-of-function mutations (stop-gain, frameshifts, etc.). However, human genetic disease can be caused by subtler mutations such as substitutions which result in a change of a single amino acid (pathogenic missense mutations). Although there are programs for estimating whether changes in amino acids are “deleterious”, they generally require a database of proteins with defined functional domains and/or a database of proteins from multiple species. Although attempts to use programs such as Polyphen-2 [38] to identify “deleterious” missense mutations in rhesus macaques have been made [39], these programs were primarily intended to be used with human data [38]. Such programs often rely, in part, on search against proteins derived from other mammals. However, we have reported that the draft rhesus macaque genome, and likely all other draft genomes, is incomplete or incorrect for approximately 50 % of all genes [24]. Hence, attempts to identify evolutionarily conserved regions within mammals have been fraught with difficulty. This may be one reason why the true impact of missense mutations scored as “deleterious” (are they truly pathogenic?) can be difficult to predict. As more mammalian genomes are brought to the same level of quality as the MacaM genome, databases which include conserved regions among mammals are likely to improve, perhaps leading to predictive programs which identify “deleterious” mutations that are truly pathogenic.

In addition to evolutionary conservation, documented association of a missense mutation with a negative phenotypic effect and variation among large numbers of humans is an invaluable source of information for determining whether or not an amino acid change is likely to be pathogenic. It is important to note that, due to species-specific differences in protein function, a variant which is pathogenic in humans is not necessarily pathogenic in rhesus macaques or other mammals. However, examination of large numbers of rhesus macaques for protein variation will likely be a fruitful strategy to determine which variants are likely to be pathogenic in this species.

## Conclusions

NGS sequences of rhesus DNA fragments captured with human exome kits can be can be aligned against the new MacaM genome and the results analyzed according to GATK best practices to identify high impact variants. Identification of heterozygous LOF mutations combined with directed breeding could be used to create rhesus macaque models of human genetic disease. This is potentially an important step in advancing translational research. This approach could also be applied to other mammalian species.

### Availability of supporting data

The exome sequence data sets supporting the results of this article are available in the Sequence Read Archive repository under accessions SRX144674 [http://www.ncbi.nlm.nih.gov/sra/SRX144674], SRX115899 [http://www.ncbi.nlm.nih.gov/sra/SRX115899], SRX144808 [http://www.ncbi.nlm.nih.gov/sra/SRX144808] and SRX145282 [http://www.ncbi.nlm.nih.gov/sra/SRX145282].

## Additional files

Additional file 1: Table S1. List of effects of mutations in four rhesus macaques (17573, ON12033, ON22186 and 002T-NHP) as annotated by SnpEff. SNV = single nucleotide variant; Indel = insertion or deletion. Effects are as follows: Intergenic = in the intergenic region. Upstream = upstream of a gene by at most 5kbp. Downstream = downstream of a gene by at most 5kbp. 5' UTR = located in the 5' UTR. 3' UTR = located in the 3' UTR. Intronic = between two exons. Splice Acceptor = two bases before exon start. Splice Donor = two bases after coding exon’s end. Splice Region = in the putative (Lariat) branch point, located in the intron. Frameshift = indel in the coding region that is not a multiple of 3. Inframe indel = indel in the coding region that is a multiple of 3. Start Lost = start codon is mutated to non-start codon. Stop gained = non-stop codon is mutated to stop codon. Stop Lost = stop codon is mutated to non-stop codon. (DOCX 116 kb) Additional file 2: Table S2. Number of variants at splice sites and percent of total splice sites for CDS and UTR exons provided for four rhesus macaques (17573, ON12033, ON22186 and 002T-NHP). (DOCX 11 kb)

## Abbreviations

ACACA: acetyl-CoA carboxylase alpha
BChE: butyrylcholinesterase
CLCA4: chloride channel accessory 4
LOF: loss-of-function
GATK: Genome Analysis Toolkit
IGV: Integrated Genome Viewer
NCBI: National Center for Biotechnology Information
NGS: next generation sequencing
RNASEL: ribonuclease L
