# Supplementary material for: Exome screening to identify loss-of-function mutations in the rhesus macaque for development of preclinical models of human disease
Source: BMC Genomics. 2016 Mar 2;17:170. doi: 10.1186/s12864-016-2509-5 (PMC4776415; doi:10.1186/s12864-016-2509-5)
Supplement: Additional file 1: Table S1. — List of effects of mutations in four rhesus macaques (17573, ON12033, ON22186 and 002T-NHP) as annotated by SnpEff. SNV = single nucleotide variant; Indel = insertion or deletion. Effects are as follows: Intergenic = in the intergenic region. Upstream = upstream of a gene by at most 5kbp. Downstream = downstream of a gene by at most 5kbp. 5' UTR = located in the 5' UTR. 3' UTR = located in the 3' UTR. Intronic = between two exons. Splice Acceptor = two bases before exon start. Splice Donor = two bases after coding exon’s end. Splice Region = in the putative (Lariat) branch point, located in the intron. Frameshift = indel in the coding region that is not a multiple of 3. Inframe indel = indel in the coding region that is a multiple of 3. Start Lost = start codon is mutated to non-start codon. Stop gained = non-stop codon is mutated to stop codon. Stop Lost = stop codon is mutated to non-stop codon. (DOCX 116 kb) [file 12864_2016_2509_MOESM1_ESM.docx]

| **SNVs per sample separated by effect** | | | | |
| --- | --- | --- | --- | --- |
|  |  |  |  |  |
|  | **17573** | **ON12033** | **ON22186** | **002T-NHP** |
| Intergenic | 74665 | 126070 | 101985 | 108881 |
| Upstream | 8039 | 13792 | 11477 | 14096 |
| Downstream | 10947 | 18861 | 15414 | 23474 |
| 5' UTR | 1655 | 3080 | 2625 | 3691 |
| 3' UTR | 4902 | 8315 | 7130 | 41263 |
| Intronic | 102736 | 175449 | 150589 | 147468 |
| Missense | 8563 | 15874 | 12416 | 13399 |
| Synonymous | 15689 | 30566 | 23145 | 25066 |
| Splice Acceptor | 16 | 18 | 22 | 21 |
| Splice Donor | 13 | 27 | 25 | 26 |
| Splice Region | 2659 | 5268 | 3916 | 4624 |
| Start Lost | 5 | 9 | 8 | 6 |
| Stop Gained | 40 | 95 | 85 | 85 |
| Stop Lost | 5 | 5 | 7 | 8 |
|  |  |  |  |  |
| **Indels per sample separated by effect** | | | | |
|  |  |  |  |  |
|  | **17573** | **ON12033** | **ON22186** | **002T-NHP** |
| Intergenic | 9739 | 16682 | 13399 | 14828 |
| Upstream | 1111 | 1853 | 1551 | 2125 |
| Downstream | 1455 | 2402 | 2063 | 3680 |
| 5' UTR | 178 | 299 | 280 | 422 |
| 3' UTR | 841 | 1443 | 1296 | 8481 |
| Intronic | 17178 | 29848 | 25697 | 24961 |
| Splice Acceptor | 11 | 38 | 24 | 37 |
| Splice Donor | 18 | 36 | 21 | 32 |
| Splice Region | 465 | 921 | 688 | 876 |
| Frameshift | 104 | 257 | 212 | 254 |
| Inframe indel | 184 | 398 | 289 | 225 |
| Start Lost | 3 | 8 | 5 | 6 |
| Stop Gained | 2 | 13 | 7 | 6 |
| Stop Lost | 0 | 4 | 3 | 3 |
|  |  |  |  |  |
| **Table description:** | List of effects of mutations as annotated by SnpEff, version 4.1h.Effects are as follows: **Intergenic** = in the intergenic region. **Upstream** = upstream of a gene by at most 5kbp. **Downstream** = downstream of a gene by at most 5kbp. **5' UTR** = located in the 5' UTR. **3' UTR** = located in the 3' UTR. **Intronic** = between two exons. **Splice Acceptor** = two bases before exon start. **Splice Donor** = two bases after coding exon's end. **Splice Region** = in the putative (Lariat) branch point, located in the intron. **Frameshift** = indel in the coding region that is not a multiple of 3. **Inframe indel** = indel in the coding region that is a multiple of 3. **Start Lost** = start codon is mutated to non-start codon. **Stop gained** = non-stop codon is mutated to stop codon. **Stop Lost** = stop codon is mutated to non-stop codon. | | | |
