# Supplementary material for: Exome screening to identify loss-of-function mutations in the rhesus macaque for development of preclinical models of human disease
Source: BMC Genomics. 2016 Mar 2;17:170. doi: 10.1186/s12864-016-2509-5 (PMC4776415; doi:10.1186/s12864-016-2509-5)
Supplement: Additional file 2: Table S2. — Number of variants at splice sites and percent of total splice sites for CDS and UTR exons provided for four rhesus macaques (17573, ON12033, ON22186 and 002T-NHP). (DOCX 11 kb) [file 12864_2016_2509_MOESM2_ESM.docx]

| Sample ID | CDS splice site mutations | UTR splice site mutations |
| --- | --- | --- |
| 17573 | 56 [.0169%] | 2 [.0117%] |
| ON12033 | 111 [.0336%] | 8 [.0468%] |
| ON22186 | 85 [.0257%] | 7 [.0409%] |
| 002T-NHP | 107 [.0324%] | 9 [.0526%] |

Additional file 2: Table S2

Number of variants at splice sites and percent of total splice sites for CDS and UTR exons

Table contains the number of variants at splice sites between introns and coding sequence exons (CDS) and untranslated region exons (UTR). This number was divided by the total number of splice sites involving CDS (330,484) or the total number splice sites involving UTR (17,098), as appropriate, to obtain the percent of exons for each of the two categories which were mutated [in brackets].
